# CYP2A6 gene polymorphism and severity of coronary atherosclerosis in Indonesian male smokers: A pilot study

**DOI:** 10.1097/MD.0000000000030308

**Published:** 2022-09-16

**Authors:** Idar Mappangara, Irawan Yusuf, Ali Aspar Mappahya, Andriany Qanitha

**Affiliations:** a Department of Cardiology and Vascular Medicine, Faculty of Medicine, Hasanuddin University, Makassar, Indonesia; b Department of Physiology, Faculty of Medicine, Hasanuddin University, Makassar, Indonesia; c Doctoral Study Program, Faculty of Medicine, Hasanuddin University, Makassar, Indonesia.

**Keywords:** atherosclerosis, CAD, CYP2A6 polymorphism, nicotine, smoking

## Abstract

Nicotine is a toxic alkaloid known to be responsible for the addictive feature of cigarettes. CYP2A6 genetic polymorphism among individuals was suspected to explain the relationship between cigarette smoking and related diseases. CYP2A6 works to slow nicotine metabolism and thus maintain a more prolonged nicotine concentration and increase nicotine exposure to the blood. We aimed to investigate the correlation between the CYP2A6 gene with the severity of coronary atherosclerosis. This cross-sectional study was conducted from April to July 2010 in Makassar Cardiac Centre, Dr Wahidin Sudirohusodo Hospital, Indonesia. Sixty-four male active smokers at the age of ≥45 years, diagnosed with coronary artery disease (CAD), were recruited and asked to smoke the usual number of cigarettes in the last 1 month prior to blood collection for CYP2A6 genotyping. Spearman correlation was performed to analyze the association between the allele variants and coronary stenosis degree, adjusted for CAD risk factors. Furthermore, we estimated the risk ratio to quantify the correlation. Of the 64 male smokers with CAD, the mean duration of smoking was 36.9 ± 8.6 years, and 49 (76.6%) were heavy smokers with >20 cigarettes per day. All 128 alleles were observed. Our results showed that all participants with CYP2A6 variants had a significant correlation with severe coronary artery stenosis (*P* = .006). Thus, this study suggests that the mutant CYP2A6 gene allele significantly increased the risk of having severe coronary stenosis 1.2 times higher compared to the wild type. This pilot study showed that CYP2A6 gene has an influential role in atherosclerotic development in male smokers. However, our findings should be confirmed with further more extensive studies.

## 1. Introduction

National Household Health Survey has shown that coronary artery disease (CAD) was the leading cause of mortality in Indonesia, counting for 26.4% of total mortality.^[1]^ Atherosclerosis, a complex mechanism involving vascular impairment leading to CAD, was started with endothelial dysfunction, promoting vasomotor dysfunction, inflammatory process, smooth muscle cell proliferation, and coagulation.^[2]^ Coronary angiography is defined as the radiographic visualization of the coronary vessels after injection of radiopaque contrast media. The angiographic characteristic of coronary artery lesion or stenosis degree was generally determined quantitatively by degree and qualitatively by coronary artery stream quality.^[3]^ Coronary stenosis of ≥70% is considered severe for the left anterior descending artery (LAD), left circumflex artery (LCx), and right coronary artery; meanwhile, a severe left main CAD was defined as >50% diameter stenosis.^[4]^

Cigarette smokers have 3 to 5 times greater risks for CAD.^[5]^ About 57,000 people die every year, and about 500,000 are suffering from various diseases because of smoking.^[6]^ Ninety per cent of nicotine or about 0.045 to 0.36 mg per cigarette will pass the blood–brain barrier and be sustained until 10 seconds.^[6]^ About 85% to 90% of nicotine was metabolized in the liver. In the human body, nicotine will be inactivated into cotinine by the enzyme Cytochrome P450 2A6 (CYP2A6) and aldehyde oxidase. At initiation, CYP2A6 will converse nicotine into the nicotine-iminium ion. Subsequently, CYP2A6 will catalyze cotinine metabolism into 3′-hydroxcotinine,5′-hydroxcotinine, or cotinine N-oxide and norcotinine. Nicotine’s half-life is only about 2 hours, but cotinine can sustain until 20 hours, and only 17% is excreted via the kidney in its unconverted form.^[7]^

Several studies reported the CYP2A6 genetic polymorphism between individuals and ethnics.^[8]^ CYP2A6 poor metabolizers or slow metabolizers (SM) will express less cotinine from nicotine; otherwise, CYP2A6 fast metabolizer (FM) metabolize nicotine faster. Subjects with the allele CYP2A6*1 metabolized nicotine almost 60%, while subjects with allele CYP2A6*4A did not metabolize nicotine.^[9,10]^ Slower nicotine metabolism may allow longer nicotine exposure and maintain longer nicotine concentration in the blood, leading to a lesser frequency of cigarette smoking.^[11]^ CYP2A6 polymorphism was reported to be existed in Indonesian population,^[12]^ however the allele frequency and variations remains unclear. This study aimed to investigate the correlation between the CYP2A6 gene variants and coronary atherosclerosis severity in Indonesian male smokers.

## 2. Methods

### 2.1. Study design and subject recruitment

This pilot cross-sectional study was conducted from April to July 2010 in Makassar Cardiac Centre, Dr Wahidin Sudirohusodo Hospital, Indonesia. Subjects were male, aged 45 years or more, an active smoker, and diagnosed with CAD (i.e., coronary angiography showed stenosis >50% in at least 1 vessel). Participants were asked to smoke the usual number of cigarettes in the last month before the blood sampling.

Participants with previous percutaneous coronary intervention, coronary artery bypass graft surgery, diabetes mellitus, coronary abnormalities, or stop smoking in the last 1 month were excluded. Active smokers were defined as people who smoke regularly and divided into mild smoker (less than half pack per day), moderate smokers (half-to-one pack per day), and severe smoker (>1 pack per day).

All participants signed a written informed consent. This study was approved by the Ethical Board Committee for Biomedical Research, Faculty of Medicine, Hasanuddin University, Makassar, Indonesia (No. 903/H4.8.4.5.31/PP36-KOMETIK).

### 2.2. Sample collection and CYP2A6 genotyping

**Figure 1.**
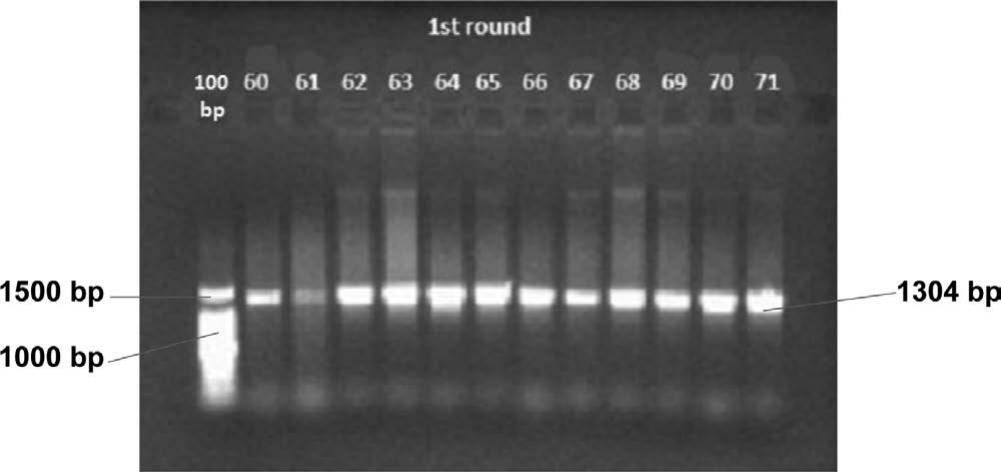
CYP2A6 genotype gel electrophoresis. Schematic RFLP used to determine the CYP2A6 genetic polymorphism. Product PCR first round with froward primer 2A6ex8F (5′-CCAGCACTTCCTGAATGAG-3′) and reverse primer 2A6R1 (5′-GCACTTATGTTTTGTGAGACATCAGAGACAA-3′). Length of product PCR are 1304 bp. This PCR product was followed by a second round PCR to determine CYP2A6*7 and CYP2A6*8. All sample amplified. PCR = polymerase chain reaction, RFLP = restriction fragment length polymorphism.

CYP2A6 genotyping with polymerase chain reaction restriction fragment length polymorphism (PCR-RFLP) method was performed in Prodia Clinical Laboratory, Jakarta, Indonesia. Briefly, blood samples were collected from a cubital vein. Genomic DNA was extracted and subjected to PCR. PCR product was triple-digested with Eco81I, AccII, and StuI restriction enzymes. The digestion patterns were determined by electrophoresis in a 2% agarose gel. The schematic RFLP patterns were used to determine the CYP2A6 genetic polymorphism as presented in Figure 1 (also available as Supplemental Digital Content, http://links.lww.com/MD/H524). The allele that encodes the phenotype most common in a particular natural population is known as the wild type (WT), while the mutant type (Mut) is having a phenotype that differs from the normal one. The mutant allele Mut/Mut is represented as SM, while the wild type WT/WT alleles as the FM.

Primers as being by Nakajima et al^[13]^ were used in this study: for CYP2A6*1 were 2A6Int8F: 5′-CAA GTG TAC CTG GCA GGA AA-3′ and 2A6reverse: 5′-TAA TTG GGT TGT TTT CTA TTG AGT-3′; for CYP2A6*4 were 2A7Int8F: 5′-CAA GTG TAT CTG GCA AGA AG-3′ and 2A6reverse: 5′-TAA TTG GGT TGT TTT CTA TTG AGT-3′; for specific allele CYP2A6*9 were 2AG_9S: 5′-GAT TCC TCT CCC CTG GAA C-3′, 2AG_9AS_WT (WT): 5′-GGC TGG GGT GGT TTG CCT TTA-3′ and 2AG_9AS_mutant (Mut): 5′-GGC TGG GGT GGT TTG CCT TTC-3′.

### 2.3. Coronary stenosis determination

Coronary stenosis was determined by computer-assisted quantitative coronary angiography using a cineangiograms angiographic device. Images collected from Left Artery Coronary were Right Anterior Oblique Projection at 30-400 (RAO 300), Left Anterior Oblique Projection at 55/600 (LAO 55/600), Left Anterior Oblique Projection at 55/600 with Cranial Angulation at 200 and Left Anterior Oblique Projection at 30-500, Caudal Angulation at 25-400 (spider position). Meanwhile, for right coronary artery, we collected images from LAO Projection at 450 with Caudal Angulation at 150 and RAO Projection position at 450. In addition, injection catheter calibration was performed before identification of the coronary artery contour, and the minimal lumen diameter was obtained using an automated edge detection algorithm.

### 2.4. Statistical analysis

Categorical variables were expressed as numbers (percentage), while continuous variables as mean ± standard deviation (SD). Baseline clinical, biochemical, and genetic characteristics were reported in total, and subsequently, comparisons were made between the WT and mutant groups. Comparisons of baseline and CAD risk factors were performed using the independent *t* test for continuous; and Pearson Chi-square or Fisher Exact test for categorical variables. Spearman correlation was performed to analyze the correlation between the allele variants and coronary stenosis degree, adjusted for CAD risk factors (i.e., hypertension, obesity, and dyslipidemia). Furthermore, we estimated the crude risk ratio (RR) to quantify the correlation.

We did avoid overfitting our models in multivariable analysis, and therefore we performed stepwise analyses in 7 adjustment models. We used the rule of thumb for multivariable analysis by having 10 to 15 outcome observations per variable in our analysis. The models are as follows:

Crude = Univariable analysis
Model 1 = Crude + Hypertension
Model 2 = Crude + Obesity
Model 3 = Crude + Dyslipidemia
Model 4 = Model 1 + Obesity
Model 5 = Model 1 + Dyslipidemia
Model 6 = Model 2 + Dyslipidemia
Model 7 = Crude + Hypertension + Dyslipidemia + Obesity

A 95% confidence interval not including one, corresponding to a 2-sided *P* value < .05, was considered statistically significant. We performed all statistical analyses with SPSS for Windows ver.16.

## 3. Results

Of the 64 male smokers with CAD (mean age 55.3 ± 8.0 years), the mean duration of smoking was 36.9 ± 8.6 years, and 49 (76.6%) were heavy smokers with >20 cigarettes per day. A total of 128 alleles were obtained. Alleles were divided into 76 (59.4%) WT and 52 (40.6%) mutant alleles. On average, participants had a normal body mass index, systolic, and diastolic blood pressure, as described in Table 1.

**Table 1:** Demographic, clinical, biochemical and genetic characteristics of the participants.

Variables	N	%	Mean	±	SD
Demographic					
Age (yr)	64	–	55.3	±	8.0
Clinical					
Systolic BP (mm Hg)	64	–	120.3	±	10.8
Diastolic BP (mm Hg)	64	–	81.7	±	9.0
BMI (kg/m^2^)	64	–	24.3	±	3.6
Biochemical					
ET-1 (ng/ml)	64	–	1.9	±	0.8
ADMA (ng/ml)	64	–	0.7	±	0.2
oxLDL (ng/ml)	64	–	208.8	±	54.6
Genetic					
*Wild type*	**76**	**59.4** *	–		–
CYP2A6*1A	42	32.8	–		–
CYP2A6*1B	34	26.6	–		–
Mutant	**52**	**40.6** *	–		–
CYP2A6*4A	21	16.4	–		–
CYP2A6*7	9	7.0	–		–
CYP2A6*8	4	3.1	–		–
CYP2A6*9	15	11.7	–		–
CYP2A6*10	3	2.3	–		–
Wild type homozygote (WT/WT)	29	45.3	–		–
Heterozygote (WT/Mut)	18	28.1	–		–
Mutant homozygote (Mut/Mut)	17	26.6	–		–
Smoking status	64	–			
Mean duration (yr)			36.9	±	8.6
Level of smoking					
<10 cigarettes/d	4	6.3	–		–
10–20 cigarettes/d	11	17.2	–		–
>20 cigarettes/d	49	76.6	–		–
Stenosis level					
Severe	59	92.2	–		–
Nonsevere	5	7.8	–		–

Categorical data were described as N (%) and continuous data as mean ± SD. Coronary stenosis of ≥70% is considered to be severe for the LA, LCx, and RCA; meanwhile, a severe left main (LM) CAD was defined as >50% diameter stenosis. Bold values indicate *P* value for trend <.0001.

ADMA = asymmetric dimethylarginine, BMI = body mass index, BP = blood pressure, ET-1 = endothelin-1, LCx = left circumflex artery, oxLDL = oxidized LDL, RCA = right coronary artery.

* *P* < .05.

Coronary angiography showed that 59 (92.2%) participants had severe coronary artery stenosis, while the remaining 5 (7.8%) participants had nonsevere stenosis. From the PCR, we found CYP2A6 allele genes as the WT (CYP2A6*1A and CYP2A6*1B) in ~60% of participants, and ~40% had a mutant allele. Of all, 45.3% have homozygote WT (WT/WT), 26.6% with mutant homozygote (Mut/Mut), and 28.1% are heterozygote (WT/Mut). Table 2 showed that the CYP2A6 gene, both of homozygote and heterozygote variants (Mut/Mut + WT/Mut), was significantly correlated with the coronary stenosis degree, both in univariable and multivariable analyses (*P* < .05).

**Table 2:** Correlation between CYP2A6 mutant gene with the severity of coronary stenosis, adjusted for cardiovascular risk factors.

Adjusted risk factors	*r*	*P* value
**Univariable analysis**	0.24	.01*
**Multivariable analyses**		
Hypertension	0.25	.004*
Obesity	0.23	.01*
Dyslipidemia	0.23	.01*
Hypertension + obesity	0.24	.01*
Hypertension + dyslipidemia	0.25	.01*
Dyslipidemia + obesity	0.21	.02*
All 3 risk factors (hypertension + dyslipidemia + obesity)	0.23	.01*

Data were analyzed using *Spearman correlation*. Hypertension was defined as systolic blood pressure ≥ 140 mm Hg, or diastolic blood pressure ≥ 90 mm Hg. Obese was defined as BMI ≥ 25 kg/m^2^. Dyslipidemia was defined as having a high plasma triglyceride, low-high-density lipoprotein (HDL) cholesterol, or increased low-density lipoprotein (LDL) cholesterol.

* *P* < .05.

From Table 3, we found no differences in cardiovascular risk factors between WT and mutant subjects. A cross-tabulation between CYP2A6 polymorphism and the severity of coronary artery stenosis was depicted in Table 4. From our analysis, the mutant CYP2A6 gene allele significantly increased the risk 1.2 times higher to have severe coronary stenosis compared to the WT.

**Table 3:** Comparison of CAD risk factors between CYP2A6 mutant and wild type.

Variables	Unit	Mutant	Wild typ*e*	*P* value
		N = 52	N = 76	
Age	Yr	56.2 ± 9.2	54.6 ± 7.0	.807
Obesity	N (%)	16 (30.8)	34 (44.7)	.113
Body mass index	kg/m^2^	24.0 ± 3.7	24.5 ± 3.5	.538
Hypertension	N (%)	12 (23.1)	28 (36.8)	.100
Systolic BP	mm Hg	121.0 ± 10.1	119.9 ± 11.3	.415
Diastolic BP	mm Hg	80.0 ± 9.7	80.0 ± 8.4	.076
Dyslipidemia	N (%)	48 (92.3)	64 (84.2)	.174
Total cholesterol	mg/dL	182.9 ± 39.4	183.2 ± 35.9	.344
HDL-cholesterol	mg/dL	36.6 ± 11.4	37.0 ± 9.0	.578
LDL-cholesterol	mg/dL	124.9 ± 34.0	124.9 ± 31.1	.192
Triglyceride	mg/dL	128.6 ± 75.0	147.3 ± 101.7	.353

Categorical data were presented as N (%), while continuous data as mean ± SD. Comparisons between groups were analyzed using *Pearson Chi-square* for categorical and *independent t test* for numerical variables.

BP = blood pressure, CAD = coronary artery disease, HDL = high-density lipoprotein, LDL = low-density lipoprotein.

**Table 4:** Cross-tabulation between CYP2A6 allele variants and coronary artery stenosis degree.

Allele variant	Stenosis degree		*P* value	RR	95% CI
	Severe	Nonsevere			
Mutant	52 (44.1)	0 (0.0)	.006*	1.15	1.07–1.29
Wild type	66 (55.9)	10 (100.0)			

Data was analyzed using *Fisher Exact test*. The 95% CI of the relative risk was calculated using *Koopman asymptomatic score*.

RR = risk ratio.

* *P* < .05.

Table 5 showed that the mutant CYP2A6 allele gene, in a combination of homozygote and heterozygote (Mut/Mut + WT/Mut) variants, was associated with an increased risk of severe coronary stenosis with RR 1.21 (95% CI 1.02–1.43) compared to the WT.

**Table 5:** Coronary artery stenosis degree differences between CYP2A6 variants (compared to wildtype).

Allele variant	Stenosis degree		*P* value	RR	95% CI
	Severe	Nonsevere			
	N = 59	N = 5			
WT/WT	24 (40.7)	5 (100.0)	Reference		
Mut/Mut	17 (28.8)	0 (0.0)	.142^*^	1.21	0.97–1.53
WT/Mut	18 (30.5)	0 (0.0)	.038^†^	–	
Mut/Mut + WT/Mut	35 (59.3)	0 (0.0)	.011^*^	1.21	1.08–1.53

Data were presented as N (%).

* Comparison was made using *Fisher Exact test* (2 × 2 table). The 95% CI of relative risk was calculated using *Koopman asymptomatic score*.

† Comparison was made using *Pearson Chi-Square* (3 × 2 table).

## 4. Discussion

This pilot study showed a significant correlation between the CYP2A6 gene allele and the severity of coronary stenosis in univariable analysis (*R* = 0.24, *P* = .01); and persistently significant, even after adjustment for potential cardiovascular risk factors (i.e. hypertension, obesity, and dyslipidemia). In the present study, we also found that all participants with the mutant variant had severe stenosis. This suggests that the mutant CYP2A6 gene allele independently increased the risk of severe coronary stenosis with RR 1.15 (95% CI 1.07–1.29, *P* = .006), compared with WT allele. Our findings are in line with a previous study, which reported that subjects with the CYP2A6*4 allele gene have a 2.16 times higher risk for CAD.^[12]^

Cigarette smoking is an irrefutable risk factor for CAD. Our previous study showed that a total of 62.3% (out of 477) CAD patients were smokers.^[14]^ In this current study, all subjects were active smokers for at least 10 years with regular smoking of ≥ 10 cigarettes per day. Although several prevention attempts have been regulated, cigarette smoking is still the number 1 cause of preventable death in developing countries.^[15]^

Increased oxidative stress and nicotine were reported to promote endothelial dysfunction.^[16]^ In a setting of cigarette smoking, free radicals may arise either from the gas or the tar phase of cigarette smoke; initiate circulating or in situ-activated macrophages and neutrophils; and plays a role as endogenous sources for reactive oxygen species, such as uncoupled endothelial nitric oxide synthase, xanthine oxidase, and the mitochondrial electron transport chain.^[2]^ The in vitro studies showed that nicotine decreased nitric oxide production and availability. Nicotine also promotes the sympathetic nervous system continuously for 24 hours, affecting vasoconstriction and coronary spasms, atherogenic for lipid profiles, as well as increasing platelet aggregation and hypercoagulation.^[11]^

Previous studies reported a higher prevalence (62.2%) of WT CYP2A6 allele gene in CAD patients, both in smoker and nonsmoker groups,^[12]^ while in our study, all CAD patients were active smokers, with ~60% WT and ~40% mutant alleles. In addition, the majority (~92%) of our CAD patients had severe coronary stenosis, which indicated that mutant variants might play an influential role in promoting severe CAD in active smokers. Our findings were supported by a previous 2-year study by Waters et al,^[17]^ which observed a narrowing of the coronary lumen diameter, 0.16 ± 0.16 mm in smokers, and 0.07 ± 0.15 mm in nonsmokers (*P* < .001).

In subjects with severe stenosis, we found predominantly (21 out of 52, 40.4%) mutant CYP2A6*4 allele genes, either homozygote (14 alleles, 41.2%) or heterozygote genotype (7 alleles, 38.9%). CYP2A6 gene alleles play a role in nicotine-metabolizing enzyme activity. Genetic mutations in this allele caused a decreasing or dismissed nicotine-metabolizing enzyme activity. WT allele CYP2A6*1 is not related to any decreased enzyme activity because the mutation occurs on the untranslated 3′-region of CYP2A6 gene; and therefore, no enzyme structural or function modification occurred.^[8,18]^ Likewise, the heterozygote (WT/Mut) CYP2A6*1A/*4 has the same metabolize rate with WT/WT homozygote.^[13]^ CYP2A6*4,*7,*8,*9, and *10 were mutant allele variants with a single amino acid mutation that led to an inactive catalytic enzyme.^[11]^ CYP2A6*4 is a form of entire deleted types, and hence, this ultimately has no enzyme activity.^[13]^ CYP2A6*9 allele contains a mutation on 5′-region in TATA box (TAGA), which changed 48T→G and decreased enzyme activity.^[17]^ CYP2A6*4/*4 homozygote (Mut/Mut) showed 15 times lower metabolizing capability compared to CYP2A6*1A.^[12]^

**Figure 2.**
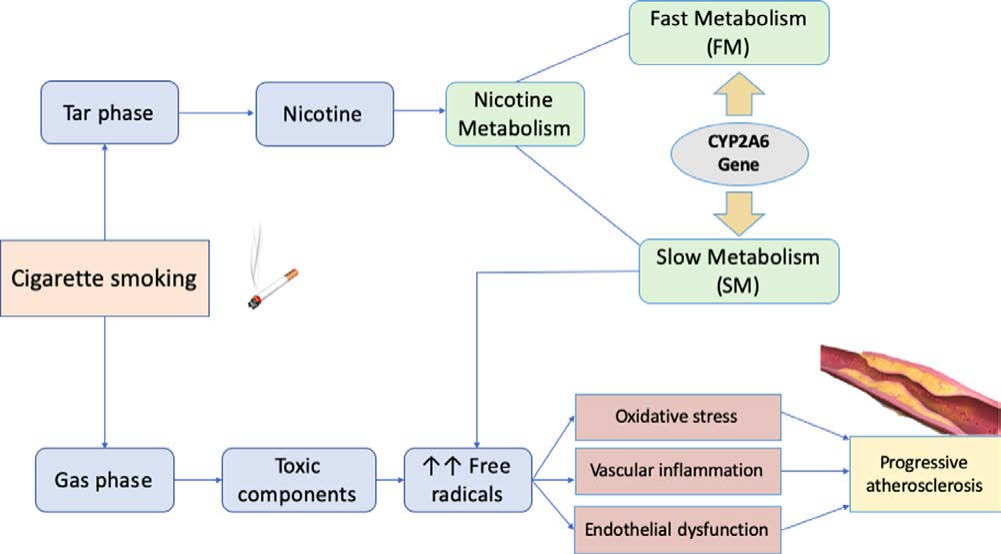
The conceptual framework on the association between CYP2A6 gene and severity of coronary atherosclerosis. CYP2A6 gene with slow metabolizers (SM) metabolize nicotine slower, while CYP2A6 fast metabolizers (FM) metabolize nicotine faster. Slower nicotine metabolism may induce longer nicotine exposures and maintain longer nicotine concentration in the blood, leading to raised free radicals. This is in turn would trigger the oxidative stress and develop a chronic low-grade vascular inflammation and endothelial dysfunction, all of which acts in the progression of the atherosclerosis.

To maintain the nicotine level, FM subjects have more cigarette consumption, but the nicotine accumulation might be diminished faster. Otherwise, in SM individuals, nicotine accumulation would stay longer, leading to an increased and more prolonged nicotine exposure to arteries, although the number of cigarettes smoked is less than FM. CYP2A6*4 is an entirely deleted type that altogether has no enzyme activity in SM type. This prolonged and high exposure to nicotine promotes endothelial dysfunction progression, followed by vasomotor dysfunction, inflammation, and vascular proliferation. All these processes escalate the progressivity of atherosclerosis, as described in Figure 2. Our study suggests that CYP2A6 gene mutation has an influential role in the severity of coronary artery stenosis, independent of the nicotine level. Mutant CYP2A6 allele, either homozygote or heterozygote, was predominantly contributed to an increased risk of severe coronary artery stenosis compared to the WT.

This present study has some limitations. Firstly, the natural design of this cross-sectional study encouraged us to measure all variables at the same point of time, and thus, whether the severity of coronary atherosclerosis was solely attributable to the CYP2A6 mutant or together with the prolonged nicotine exposure, remains unclear. Secondly, we could not consistently detect the correlation between CYP2A6 polymorphism and severe stenosis in homozygote (Mut/Mut) subjects. The correlation was unstable, but the risk ratio was increased, and thus, this may reflect the limited power from our sample size to perform further analysis. Finally, in this study, all our participants were active smokers with at least 10-year exposure to cigarette smoking, and hence, it is predictable that the majority of our participants could have severe stenosis. As this pilot study has a relatively small sample size of Indonesian male smokers, generalization to a broader population should be considered carefully, and thus, further large and prospective studies that involving both sexes and different levels of smoking are needed to confirm our findings and to establish robust conclusions on how much smoking contributes to the development of CAD. Although the small number of nonsevere stenosis restricted us to perform subgroup analysis, we still captured significant correlations. In the future, the potential mechanisms that underlie the correlation should be explored in larger prospective or interventional studies.

## 5. Conclusions

CYP2A6 gene polymorphism has a significant correlation with the severity of coronary atherosclerosis in male smokers, where the mutant CYP2A6 independently increased the risk of having severe coronary stenosis. Our findings may be of particular relevance for early prevention of CAD in this population, however, further large studies are needed to confirm these findings.

## Acknowledgements

The authors gratefully acknowledge the staff of PRODIA Laboratory for all their contributions to this manuscript.

## Author contributions

IM, AAM, and IY conceived the idea and formulated the research questions. IM performed the literature searching and prepared the initial manuscript. IM and AQ performed data analyses. AQ made critical revisions and provided the conceptual framework. AAM and IY reviewed and advised further revisions. All authors read and approved the final manuscript.

**Conceptualization:** Ali Aspar Mappahya, Idar Mappangara, Irawan Yusuf.

**Data curation:** Idar Mappangara, Irawan Yusuf.

**Formal analysis:** Andriany Qanitha, Idar Mappangara.

**Funding acquisition:** Idar Mappangara.

**Investigation:** Idar Mappangara.

**Methodology:** Ali Aspar Mappahya, Andriany Qanitha, Irawan Yusuf.

**Project administration:** Idar Mappangara.

**Resources:** Andriany Qanitha, Idar Mappangara, Irawan Yusuf.

**Software:** Andriany Qanitha.

**Supervision:** Ali Aspar Mappahya, Irawan Yusuf.

**Validation:** Ali Aspar Mappahya, Irawan Yusuf.

**Visualization:** Andriany Qanitha.

**Writing – original draft:** Andriany Qanitha, Idar Mappangara.

**Writing – review & editing:** Andriany Qanitha, Idar Mappangara, Irawan Yusuf.

## Supplementary Material


