# Supplementary material for: CYP2A6 gene polymorphism and severity of coronary atherosclerosis in Indonesian male smokers: A pilot study
Source: Medicine (Baltimore). 2022 Sep 16;101(37):e30308. doi: 10.1097/MD.0000000000030308 (PMC9478272; doi:10.1097/MD.0000000000030308)
Supplement: Supplementary file 1 [file medi-101-e30308-s001.pdf]

Untuk Diagnosa Lebih Baik
Dengan Layanan Sepenuh Hati

Laboratorium Riset & Esoterik
Laboratorium Klinik Prodia
Jl. Kramat Raya No. 150, lt 3, Jakarta 10430
Telp. (021) 314 4182, Fax. (021) 314 4181

HASIL PEMERIKSAAN
Analisa Gen CYP2A6
Sampel Penelitian dr. Idar Mappangara (Makassar)

Hasil Gel Elektroforesis

CYP2A6 *1/*4

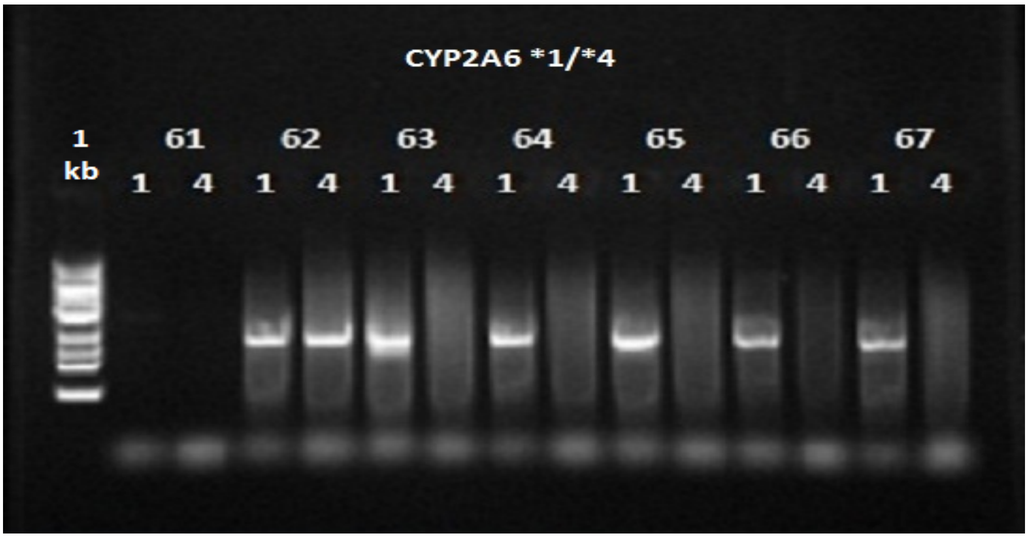

Produk PCR	CYP2A6	1937 bp
	CYP2A7	1936 bp

Hasil RFLP *AccII*

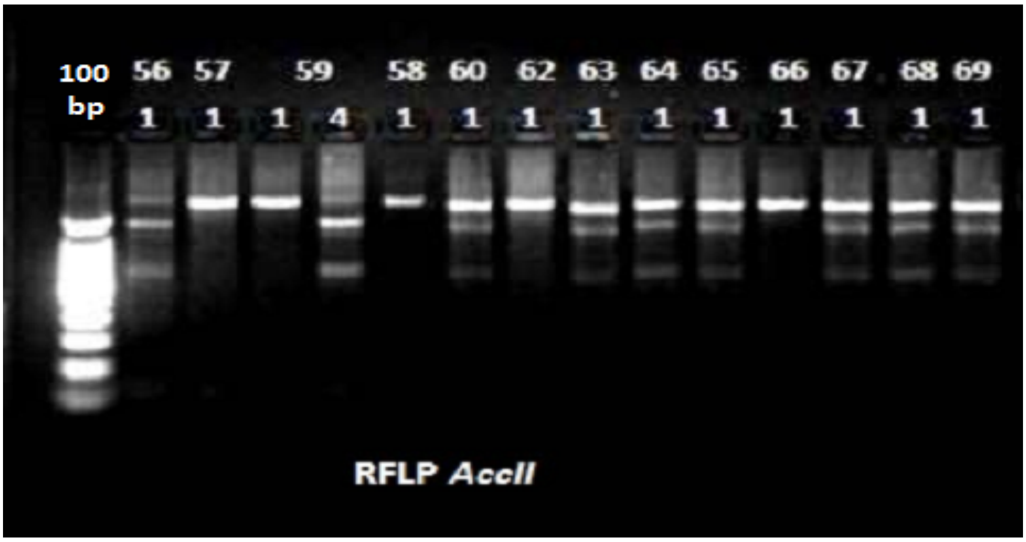

Hasil 1st Round

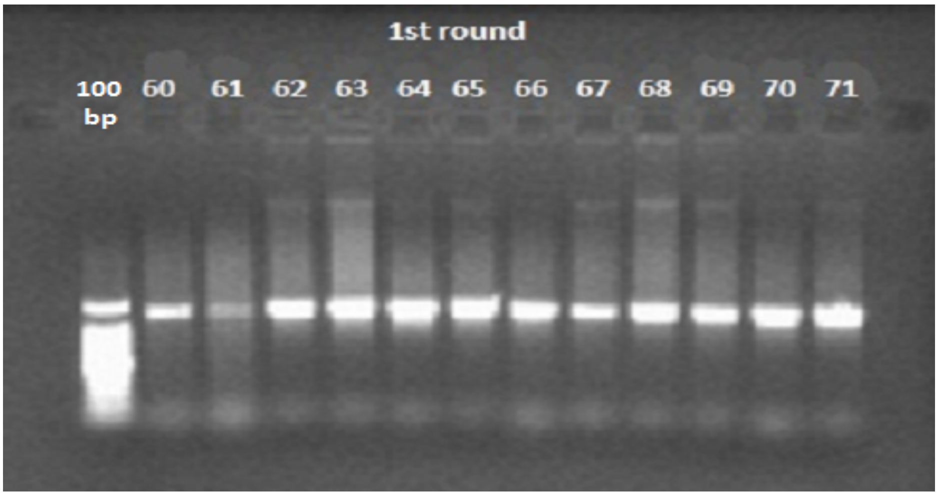

Produk PCR 1304 bp

Hasil 2nd Round

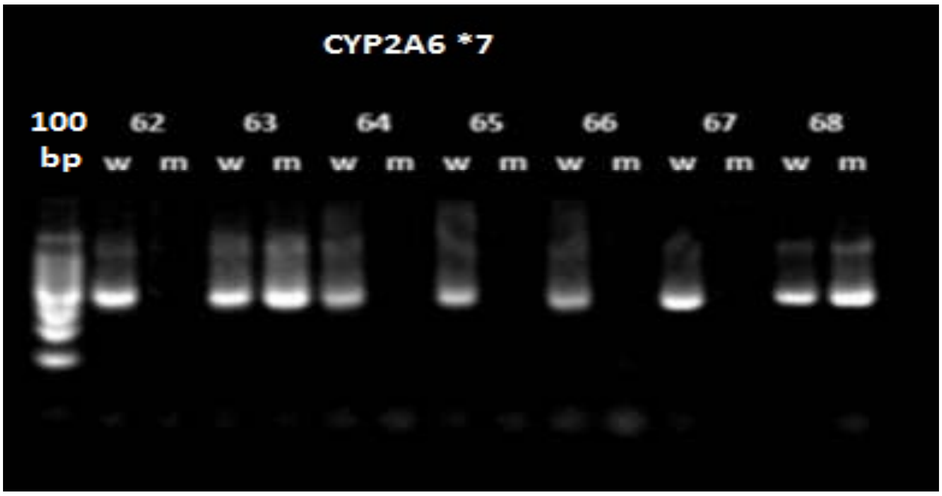

Produk PCR CYP2A6 *7 394 bp

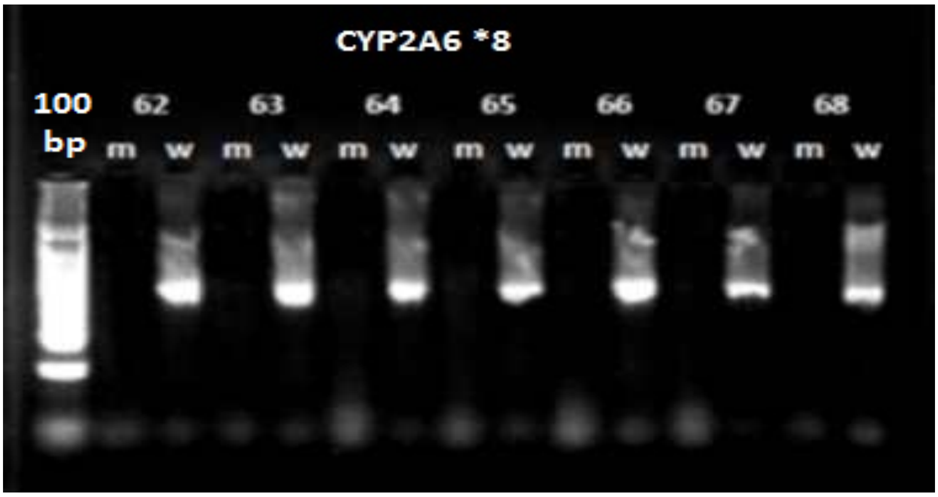

Produk PCR CYP2A6 *8 394 bp

Hasil CYP2A6 *9

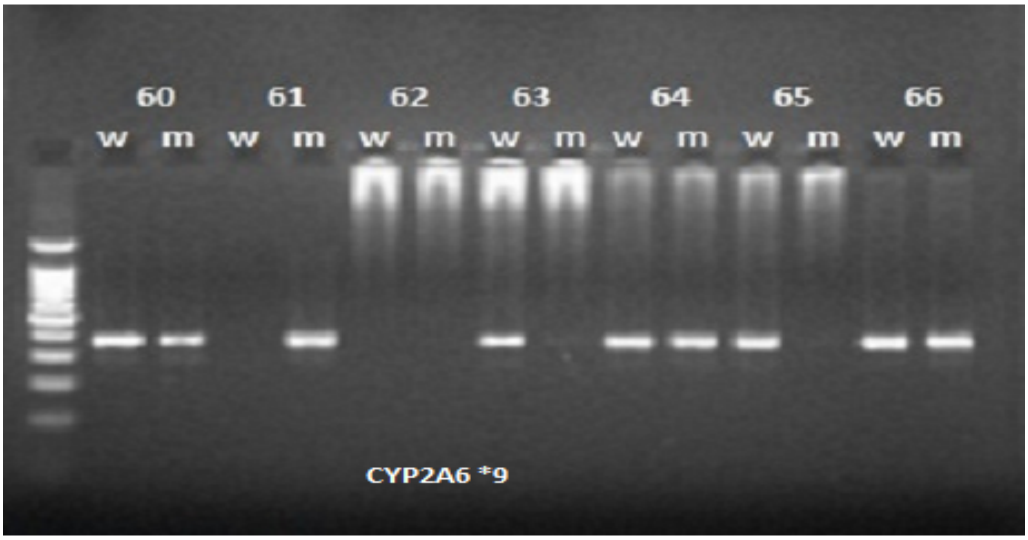

Produk PCR CYP2A6 *9 368 bp

Keterangan:

w : wild type

m : mutan

Jakarta, 26 April 2012

PT. PRODIA WIDYAHUSADA

(Miswar Fattah, M.Si)

Research & Esoteric Test Laboratory Head
